# Atomic Force Microscopy Provides New Mechanistic Insights into the Pathogenesis of Pemphigus

**DOI:** 10.3389/fimmu.2018.00485

**Published:** 2018-03-28

**Authors:** Franziska Vielmuth, Volker Spindler, Jens Waschke

**Affiliations:** Institute of Anatomy, Faculty of Medicine, Ludwig-Maximilians-Universität München, Munich, Germany

**Keywords:** atomic force microscopy, desmosome, pemphigus, desmosomal cadherin, cell adhesion

## Abstract

Autoantibodies binding to the extracellular domains of desmoglein (Dsg) 3 and 1 are critical in the pathogenesis of pemphigus by mechanisms leading to impaired function of desmosomes and blister formation in the epidermis and mucous membranes. Desmosomes are highly organized protein complexes which provide strong intercellular adhesion. Desmosomal cadherins such as Dsgs, proteins of the cadherin superfamily which interact *via* their extracellular domains in Ca^2+^-dependent manner, are the transmembrane adhesion molecules clustered within desmosomes. Investigations on pemphigus cover a wide range of experimental approaches including biophysical methods. Especially atomic force microscopy (AFM) has recently been applied increasingly because it allows the analysis of native materials such as cultured cells and tissues under near-physiological conditions. AFM provides information about the mechanical properties of the sample together with detailed interaction analyses of adhesion molecules. With AFM, it was recently demonstrated that autoantibodies directly inhibit Dsg interactions on the surface of living keratinocytes, a phenomenon which has long been considered the main mechanism causing loss of cell cohesion in pemphigus. In addition, AFM allows to study how signaling pathways altered in pemphigus control binding properties of Dsgs. More general, AFM and other biophysical studies recently revealed the importance of keratin filaments for regulation of Dsg binding and keratinocyte mechanical properties. In this mini-review, we reevaluate AFM studies in pemphigus and keratinocyte research, recapitulate what is known about the interaction mechanisms of desmosomal cadherins and discuss the advantages and limitations of AFM in these regards.

## Introduction

Pemphigus with the two main forms pemphigus vulgaris (PV) and pemphigus foliaceus (PF) represents a group of autoimmune blistering skin diseases in which autoantibodies develop primarily against the desmosomal cadherins desmoglein (Dsg) 1 and 3. This leads to weakened keratinocyte cohesion by a vast and yet only partially understood set of mechanisms and in consequence causes intraepidermal splitting (1, 2). Patients suffer from painful blistering affecting skin and mucous membranes, including the risk of infections and nutritive problems (3, 4). A broad range of methods, including functional adhesion assays, molecular biology, and immunological approaches as well as animal models are used study pemphigus pathogenesis (2, 5). The investigation of some mechanisms underlying desmosome dysfunction, e.g., impaired desmosome turnover, requires complex model systems such as passive IgG transfer in mouse models or ultrastructural analysis of human skin *ex vivo* (6). However, reductionist approaches such as atomic force microscopy (AFM) analysis of Dsg-binding properties and distribution either in cell-free models or 2D keratinocyte cultures yield important information about the effects of autoantibodies on the function of cell adhesion molecules. Moreover, these effects can be analyzed in concert with morphological alterations typical for pemphigus such as keratin filament retraction and changes in overall mechanical properties of keratinocytes. Insights in the mechanisms of desmosomal cadherin interactions and their regulation by intracellular signaling and plaque proteins may provide the molecular basis for targeted therapies in pemphigus. In the following, we will summarize the conclusions that could be drawn from studies utilizing AFM force spectroscopy and elasticity mapping to investigate pemphigus pathogenesis and outline strengths and weaknesses of this experimental approach.

## Principle of Cell-Free and Cell Surface AFM Measurements

**Figure 1:**
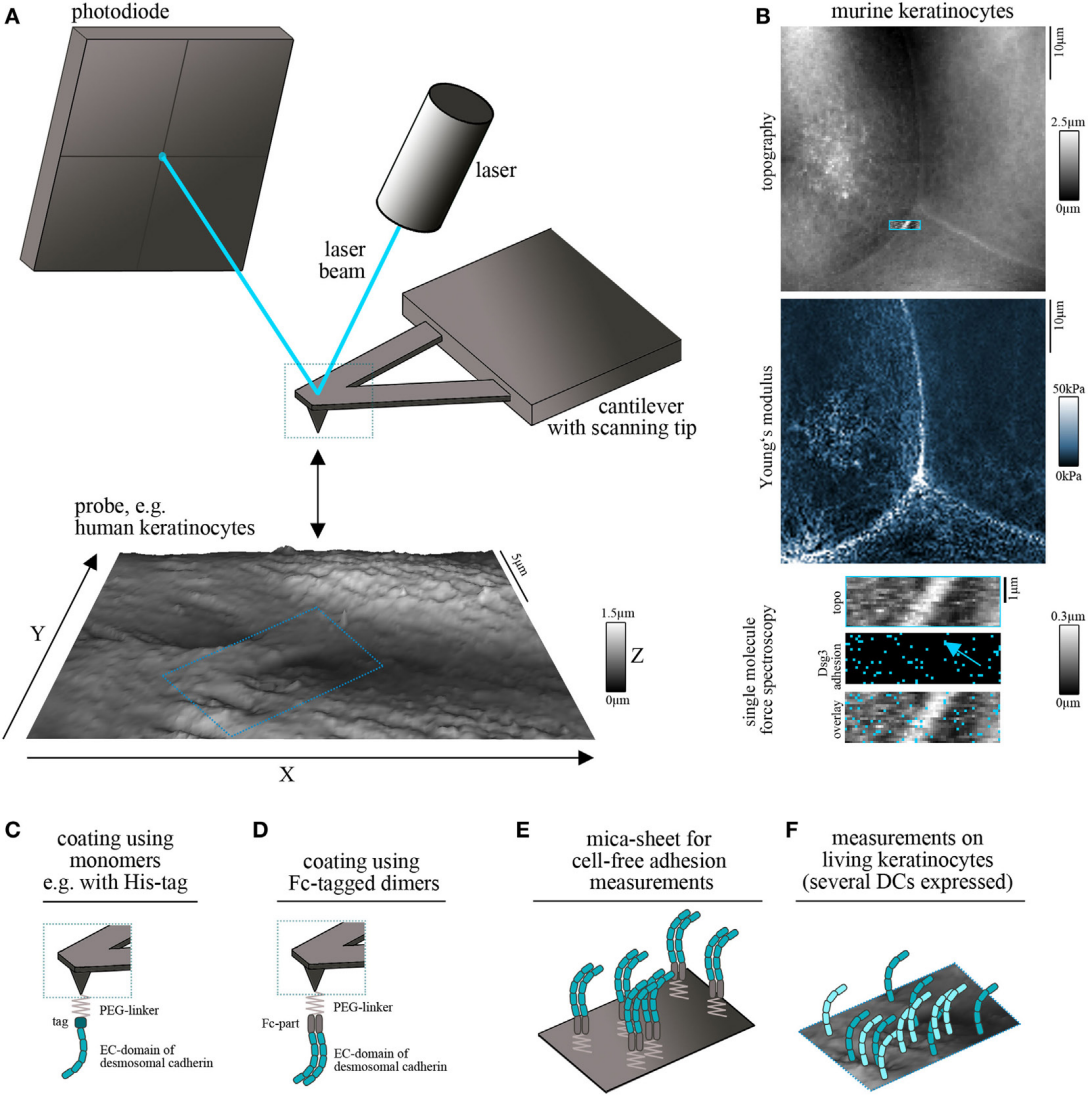
Atomic force microscopy (AFM) setup for cadherin binding studies. **(A)** Schematic of an AFM setup. A flexible cantilever equipped with a sharp tip is repetitively lowered to and retracted from the surface of the probe. Deflection of the cantilever while contacting the surface is detected by a laser pointed on the cantilever and provides information about surface topography and mechanical properties. **(B)** Example for simultaneous measurement of topography (with elevated cell borders and filamental structures on the cell surface), elasticity (Young’s modulus) and Dsg3 adhesion map (with each blue pixel represents on Dsg3-dependent binding event, arrow points on the cell border) on living murine keratinocytes. **(C,D)** To study single-molecule interaction tips can be functionalized with recombinant adhesion molecules using PEG-linkers. For desmosomal cadherins coating was conducted using full-length extracellular domains as either monomers **(C)** or Fc-tagged dimers **(D)**. **(E)** Probe setup for cell-free measurements on mica sheets coated with Fc-tagged dimers of desmosomal cadherin extracellular domains. **(F)** Probe setup for measurements on living keratinocytes. Cells express several desmosomal cadherin isoforms on their cell surface.

Atomic force microscopy is used to construct topography maps based on the deflections of a flexible cantilever equipped with a sharp detection tip. Driven by highly accurate piezo steppers, the tip scans a freely definable region of interest while deflection of the cantilever is detected by the displacement of a laser beam on a photodiode (Figure 1A). This setup allows the measurement of virtually all kinds of materials. Being a non-optical imaging technique, the resolution is not limited by diffraction of light and reaches a spatial resolution down to 0.5–1 nm (7). Important for the field of basic biology, living cells, e.g., keratinocytes, can be imaged under near-physiological conditions (37°C, medium) without the necessity of fixation (8, 9). Depending on the imaging mode, mechanical properties such as elasticity of the sample can be acquired together with information about the surface topography (10, 11) (Figure 1B). The combination of AFM topography and elasticity mapping with force spectroscopy provides an additional set of data that can be extracted from the same scan (Figure 1B). In this approach, recombinant adhesion molecules, e.g., the extracellular domains of desmosomal cadherins, are coupled to the AFM tip (Figures 1C,D). The tip is repetitively lowered to and retracted from a given surface, e.g., a cell membrane. Scanning with these functionalized tips provides information about the binding partners of the respective molecule, their localization (e.g., position in the membrane) (Figure 1B), and a set of biophysical properties of single-molecule interactions, such as binding forces, lifetimes of the respective bonds, and step position (12, 13).

Several methods are established for protein functionalization of AFM cantilevers (14). However, usage of heterobifunctional PEG-linkers is often preferred because it allows coupling of the molecule of interest to the distal end of the linker and ensures a reproducible detection radius throughout the experiments (15). In addition, these linkers allow coupling with a broad range of molecules through amino groups (15). Thus, it is possible to coat full-length extracellular domains of desmosomal cadherins which has been done with his-tagged monomers (Figure 1C) (16) as well as with Fc-tagged dimers (Figure 1D) (17). The second setup was applied based on experiments using classical cadherins, in which cis-dimerization was thought to be crucial for proper adhesive function (18, 19). However, both approaches showed specific homophilic and heterophilic binding events (16, 20–22). Due to the freely moving linkers, the achievable resolution is reduced to around 50 nm (14) which is suitable for capturing desmosomal cadherin clusters at the surface of living keratinocytes. Importantly, AFM force spectroscopy can be combined with other imaging modalities. These range from conventional and superresolution fluorescence microscopy techniques to electron microscopy and may help to overcome technical limitations, such as non-specificity of adhesion measurements and low imaging speed (23, 24). Together, this highly flexible AFM-based multimodal imaging allows the simultaneous acquisition of a wide range of different parameters.

For characterization of binding properties of desmosomal cadherins often cell-free approaches are used in which recombinant proteins are immobilized not only on the scanning tip but also on the surface of, e.g., a silicon nitrite mica-sheet (25). This reductionist model allows unequivocal evaluation of binding partners and forces because the possible interaction partners are clearly defined (Figure 1E). By contrast, keratinocytes express several isoforms of desmosomal cadherins (Figure 1F) which hinders a clear identification of interaction partners. Moreover, cell monolayers are more complicated to handle because of the necessity of measurements under near-physiological conditions, including temperature control and application of media to avoid starving (8). The continuous reorganization and morphological changes of the monolayer limit lateral resolution and are challenging because of the time necessary for AFM measurements (14). On the other side, the increased complexity by application of living cells has numerous advantages and adds novel possibilities to characterize desmosomal adhesion. Changes of cell topography and mechanical properties can be monitored in response to manipulation of signaling pathways or genetic depletion of specific proteins (26–28). In addition, alterations in the localization of Dsg clusters at the surface of living keratinocytes, their mobility (20, 29), and the binding properties can be elucidated (20, 30). *Vice versa*, changes in cell behavior or intracellular signaling activity can be detected following AFM-based manipulation such as indentation of the membrane or severing of cytoskeletal components (31, 32).

## AFM to Elucidate Dsg-Binding Partners and to Study the Effects of Autoantibodies

Desmogleins and desmocollins have been shown to bind both in homophilic- and heterophilic fashion under cell-free conditions (17, 33, 34). By cell-free single-molecule AFM force spectroscopy using recombinant Fc-dimers of the entire extracellular domain, we found that Dsg1, Dsg2, Dsg3, and Dsc3 can interact homophilically. Importantly, these homophilic interactions were blocked by both EGTA treatment as well as incubation with specific antibodies (16, 20, 21, 35, 36). As another indication for specific homophilic interactions, the bond rupture forces increased with the applied loading rate similar to classical cadherins (18, 37–39). Corresponding lifetimes were delineated at τ_0_≈0.17 for Dsg1, τ_0_≈0.31 for Dsg3, and τ_0_≈0.24s for Dsc3 in cell-free AFM experiments and τ_0_≈0.31 for Dsg3-dependent binding events on murine keratinocytes (20, 29, 35, 36) which were significantly lower than detected for classical cadherins (18, 37). Homophilic interactions of Dsc2 but not Dsg2 monomers were also observed recently (16). With regard to heterophilic interactions, binding of Dsg2 to Dsc2 and Dsg3 was observed by AFM as well as interactions between Dsg1 and Dsc3 (16, 20, 35). In a systematic approach, only heterophilic interactions of Dsgs and desmocollins were found by surface plasmon resonance measurements although homophilic interactions were observed when high concentrations of molecules were applied, which allowed to determine the crystal structure of two interacting Dsg2 molecules (40). The reasons for these in part contradictory *in vitro* findings are unclear yet. Nevertheless, in living keratinocytes, homophilic interactions appear to be a primary mode of interaction as revealed by extracellular cross-linking (41). In line with this, we detected primarily homophilic interactions of Dsg3 on the surface of living keratinocytes (20, 29).

**Figure 2:**
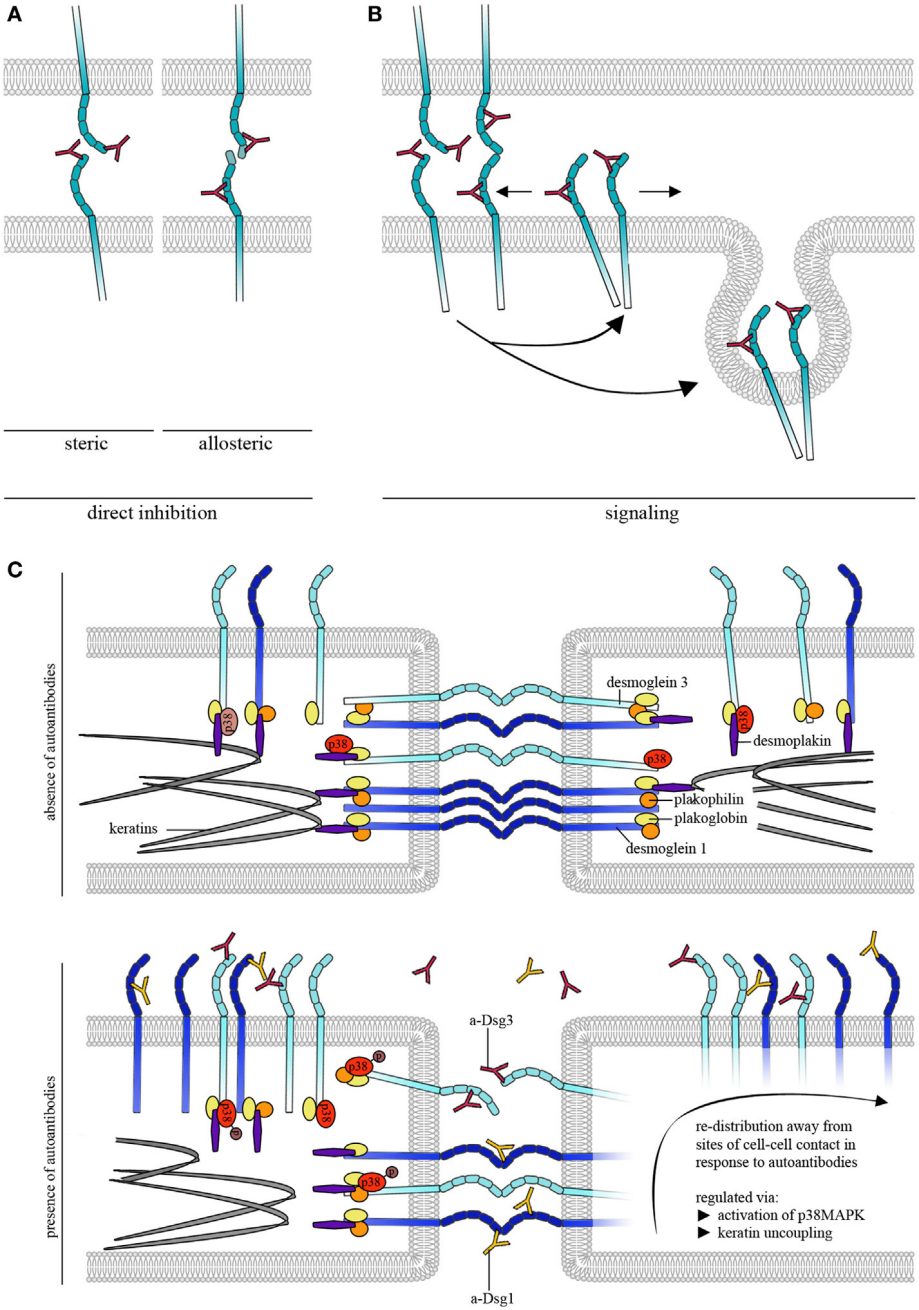
Autoantibody effects on desmosomal cadherin binding properties. **(A)** To cause direct inhibition of desmoglein (Dsg)-binding autoantibodies may either sterically hinder desmosomal cadherin interaction by preferentially targeting the adhesive EC1 domain or allosterically lead to conformational changes, which also may involve the adhesive interface. **(B)** Autoantibodies induce intracellular signaling leading to reorganization and internalization of Dsg molecules. **(C)** Schematic of autoantibody effects on desmosomal cadherin distribution. For simplification, only Dsgs are shown. Under control conditions, Dsg3 is uniformly distributed over the cell surface whereas Dsg1 shows dense clustering along cell–cell contacts. Binding of pathogenic autoantibodies causes direct inhibition of Dsg3 but not of Dsg1 interactions. For both, Dsg1 and 3, binding of autoantibodies induces redistribution of binding events away from cell–cell contact sites, a process that is likely regulated through by keratin uncoupling and activation of p38MAPK.

Atomic force microscopy was further used to study the effects of pemphigus autoantibodies on Dsg binding. To be pathogenic and result in loss of cell cohesion, autoantibodies would either need to block Dsg interactions or lead to reorganization and internalization of Dsg molecules (Figure 2A). In the first concept, autoantibodies may sterically hinder interaction by preferentially targeting the adhesive EC1 domain of Dsgs or allosterically lead to conformational changes of the adhesive interface. These modes of interactions may be summarized as direct inhibition (Figure 2A). Release of Dsgs from desmosomes and endocytosis would require additional cellular mechanisms and intracellular signaling (Figure 2B). For instance, it is possible that extradesmosomal molecules serve as scaffolds which, dependent on autoantibody binding modulate signaling pathways and, thus, influence the composition and turnover of desmosomes. We sought to apply pemphigus autoantibodies in cell-free AFM in order to demonstrate direct inhibition of Dsg binding which represented the most likely mechanism of antibody action (42). However, we were surprised that PF-IgG did not directly interfere with Dsg1 binding (17). Meanwhile, using various IgG fractions of both PF-IgG and PV-IgG, we were not able to detect direct inhibition of Dsg1 interaction, both in cell-free and in cell-based measurements (34, 43, 44). By contrast, in all experiments using PV-IgG or the monoclonal Dsg3-specific antibody AK23 derived from a pemphigus mouse model (45) direct inhibition of Dsg3 binding was demonstrated (26, 34, 43, 44, 46). Thus, these data do not rule out that some autoantibodies targeting Dsg1 may occur which also cause steric hindrance, especially since some antibodies isolated from patients by phage display have been shown to interact with both Dsg1 and Dsg3 (47). However, the amount of these appears to be low if present in IgG fractions of many patients.

Atomic force microscopy studies also showed that a Dsg-specific tandem peptide designed to cross-link interacting Dsgs was effective to abrogate PV-IgG- and AK23-induced loss of keratinocyte cohesion in culture and in living mice indicating that direct inhibition of Dsg3 binding contributes to blister formation (46, 48). However, since the peptide approach also abolished activation of pathogenic signaling pathways such as p38MAPK and pharmacologic inhibition of p38MAPK was effective to override autoantibody-induced loss of cell adhesion in the presence of autoantibodies directly inhibiting Dsg3 interaction, steric hindrance alone appears not to be sufficient to cause full loss of keratinocyte cohesion (26, 48). It has to be noted that a limitation of AFM interaction studies is that recombinant Dsg molecules attached to the AFM probe primarily will interact with extradesmosomal Dsg rather than Dsg1 and 3 in the core of desmosomes. This means that AFM studies cannot definitely prove that PV-IgG and AK23 are directly inhibiting the interaction of Dsg3 molecules inside of desmosomes. Nevertheless, because it was shown by immune-electron microscopy that the IgG-variant but not the IgM-variant of AK23 accessed the desmosomal core (49), it is likely that direct inhibition of Dsg3 binding in pemphigus also occurs in desmosomes and thereby contributes to desmosome dysfunction (6).

## AFM to Quantify Alterations of Keratinocyte Elasticity and Cytoskeletal Reorganization in Response to Autoantibodies

The cytoskeleton of eukaryotic cells comprises actin filaments, intermediate filaments, and microtubules. Keratin filaments, the intermediate filaments in cells of epithelial origin anchoring desmosomes, are pronouncedly affected in pemphigus. The so called “keratin retraction” describes the uncoupling of keratin filaments from desmosomes and the clustering around the nucleus. By AFM, keratin filaments can be visualized as stiff bundles underneath the membrane in the cell periphery inserting perpendicular to areas of cell–cell contact into desmosomes (26, 28). Interestingly, changes in the peripheral keratin network occur very rapidly within 1 h after autoantibody incubation and appear to even precede Dsg3 endocytosis, another hallmark of pemphigus (26, 50). Here, the first visible alteration is a reduced amount of keratin filaments in the basal part of the cell near areas of cell–cell contact, a region implicated in assembly of keratin filaments (51).^1^ Furthermore, as demonstrated by AFM elasticity mapping and optical stretching, keratin filaments in contrast to the actin cytoskeleton are the main constituents responsible for keratinocyte stiffness (52–54). In line with these data and the observations that keratin filaments are rapidly altered upon application or pemphigus autoantibodies, elasticity changes in response to autoantibody binding were described (31). By probing the membrane above the nucleus, a rapid reduction in cellular stiffness was observed within minutes followed by an increase after several hours, the latter of which may be a result of keratin clustering around the nucleus. Nevertheless, it is unclear whether these changes are mainly a result of keratin reorganization or whether other mechanisms contribute, as these changes were related to FasL-dependent apoptotic signaling (31).

The actin cytoskeleton is also severely altered by autoantibody binding (55, 56). Strengthening of the cortical actin meshwork prevented autoantibody-induced loss of cell cohesion in keratinocytes, indicating a contribution of actin reorganization to epidermal blistering (57). In principle, the actin meshwork underneath the membrane is accessible for AFM-based imaging (58–60), albeit with reduced resolution compared to approaches involving a membrane “deroofing” step or inside-out measurements of membrane patches (61, 62). By AFM, the differentiation between the cortical actin and other cytoskeletal components is barely possible, especially because a cortical keratin filament meshwork may exist (63). Nevertheless, the observation that the delicate mesh pattern of the membrane of murine keratinocytes appears similar in cells with and without expression of keratin filaments indicates that these structures represent the cortical actin cytoskeleton (20). If and in which timeframe specific changes of the cortical actin cytoskeleton can be detected by AFM imaging remains to be elucidated.

## Studies on Dsg Distribution Patterns and Binding Properties Modulated by Pemphigus Antibodies

In keratinocytes, Dsg3 and 1 are differentiation-dependently localized at sites of cell–cell junctions (64, 65). Interestingly, AFM measurements showed that Dsg3 binding events are uniformly distributed on the surface of living keratinocytes (29) (Figure 2C). This could be related to Dsg3 molecules that are detectable away from junctions and referred to as extradesmosomal (30, 66, 67). Thus, molecules detected close to cell–cell junctions may be extradesmosomal, located in desmosomal precursors or located at the edge of the desmosomal core, as the “center” of the tightly packed desmosomes is most likely not accessible for the AFM scanning tip. Nevertheless, these molecules differ with regard to their binding properties. Molecules at cell–cell junctions reveal higher binding forces compared to molecules on the cell surface above the nucleus (29). By contrast, Dsg1 binding events are not distributed uniformly but rather show higher binding frequencies along cell–cell junctions, indicating that clustering of Dsgs differs between isoforms (44, Figure 2C). In this context, keratins not only regulate cell mechanics (53, 54) but also differentially regulate Dsg-binding properties. For example, keratins are crucial for maintenance of Dsg3 binding strength as well as for distribution of Dsg1 at cell junctions (20, 44).

Binding of pemphigus autoantibodies to the extracellular domains of Dsg1 and 3 on the surface of living keratinocytes was shown to induce altered clustering of the targeted molecules (68–70). Using AFM, redistribution of Dsg1 binding events away from cell junctions occurred after treatment with pemphigus IgG fractions containing a-Dsg1 antibodies which may explain the structural changes described above (44). Dsg1 redistribution seems to be dependent on uncoupling from the keratin filaments which is a common phenomenon after treatment with pathogenic autoantibodies and precedes reduction of Dsg1 binding strength (Figure 2C) (44, 48, 55). Due to direct inhibition of Dsg3 interaction, redistribution of Dsg3 molecules could not be evaluated after autoantibody treatment (26, 34). However, modulation of signaling pathways such as p38MAPK was used to mimic some effects of pemphigus autoantibodies on Dsg3 binding properties. p38MAPK is a central signaling molecule in pemphigus which is activated by binding of autoantibodies and was demonstrated to form a signaling complex containing Dsg3 and Dsc3 (71). Furthermore, activation of p38MAPK has been linked to keratin retraction and Dsg internalization whereas inhibition of p38MAPK prevented loss of intercellular adhesion (Figure 2C) (55, 66, 71–73). Interestingly, activation of p38MAPK led to keratin retraction and redistribution of Dsg1 and 3 binding events away from junctions indicating that p38MAPK signaling participates in the regulation of Dsg clustering (Figure 2C) (44). Furthermore, inhibition of p38MAPK prevented autoantibody-induced redistribution of Dsg1 binding events and restored Dsg3 binding strength under conditions where keratinocytes were depleted from keratins (20, 44) indicating that Dsg-binding properties are strongly dependent on p38MAPK. Taken together, AFM adds important information on molecule distribution and binding properties of Dsgs after autoantibody incubation.

## Conclusion

Atomic force microscopy complements a broad range of methods in pemphigus research. Under cell-free conditions, AFM enables characterization of single-molecule desmocadherin interactions with and without the presence of pemphigus autoantibodies. When applied on living keratinocytes, this can be complemented by monitoring cytoskeletal alterations. So far, AFM is the only technique with which direct inhibition of Dsg interactions by pemphigus autoantibody binding was detected on the single-molecule level. Furthermore, it provides insights into alterations of keratinocyte properties. Although interaction partners on living cells cannot be completely identified and temporal resolution is low compared to other live-cell imaging approaches, investigation of Dsg mobility and redistribution in response to autoantibodies may add important information about the underlying mechanisms of loss of cell cohesion in pemphigus. Similarly, combination of Bio-AFM with high-resolution imaging techniques such as STED microscopy may elucidate whether alterations of Dsg binding properties in response to autoantibody binding maybe mediated by different association with signaling molecules and plaque proteins.

## Author Contributions

FV, VS, and JW designed the review, evaluated the literature, and wrote the manuscript.

## Conflict of Interest Statement

The authors declare that the research was conducted in the absence of any commercial or financial relationships that could be construed as a potential conflict of interest. The reviewer PK and handling Editor declared their shared affiliation.

## Footnotes

^1^Schlögl E, Radeva M, Vielmuth F, Schinner C, Waschke J, Spindler V. (under review). Keratin retraction and Dsg3 internalization independently contribute to autoantibody-induced cell dissociation in pemphigus vulgaris. Submitted to Immunology.

## References

[1] Kasperkiewicz M Ellebrecht CT Takahashi H Yamagami J Zillikens D Payne AS Pemphigus. Nat Rev Dis Primers (2017) 3:17026. 10.1038/nrdp.2017.26 28492232 PMC5901732

[2] Spindler V Eming R Schmidt E Amagai M Grando S Jonkman MF Mechanisms causing loss of keratinocyte cohesion in pemphigus. J Invest Dermatol (2018) 138(1):32–37. 10.1016/j.jid.2017.06.022 29037765

[3] Kneisel A Hertl M. Autoimmune Bullous skin diseases. Part 2: diagnosis and therapy. J Dtsch Dermatol Ges (2011) 9(11):927–47. 10.1111/j.1610-0387.2011.07809.x 22026362

[4] Kneisel A Hertl M Autoimmune Bullous skin diseases. Part 1: clinical manifestations. J Dtsch Dermatol Ges (2011) 9(10):844–56; quiz 57. 10.1111/j.1610-0387.2011.07793.x 21955378

[5] Pollmann R Eming R Research techniques made simple: mouse models of autoimmune blistering diseases. J Invest Dermatol (2017) 137(1):e1–6. 10.1016/j.jid.2016.11.003 28010761

[6] Spindler V Waschke J Pemphigus – a disease of desmosome dysfunction caused by multiple mechanisms. Front Immunol (2018) 9:136 10.3389/fimmu.2018.00136 29449846 PMC5799217

[7] Li M Dang D Xi N Wang Y Liu L. Nanoscale imaging and force probing of biomolecular systems using atomic force microscopy: from single molecules to living cells. Nanoscale (2017) 9(45):17643–66. 10.1039/c7nr07023c 29135007

[8] Allison DP Mortensen NP Sullivan CJ Doktycz MJ. Atomic force microscopy of biological samples. Wiley Interdiscip Rev Nanomed Nanobiotechnol (2010) 2(6):618–34. 10.1002/wnan.104 20672388

[9] Parot P Dufrene YF Hinterdorfer P Le Grimellec C Navajas D Pellequer JL Past, present and future of atomic force microscopy in life sciences and medicine. J Mol Recognit (2007) 20(6):418–31. 10.1002/jmr.857 18080995

[10] Alonso JL Goldmann WH. Feeling the forces: atomic force microscopy in cell biology. Life Sci (2003) 72(23):2553–60. 10.1016/S0024-3205(03)00165-6 12672501

[11] Zlatanova J Lindsay SM Leuba SH. Single molecule force spectroscopy in biology using the atomic force microscope. Prog Biophys Mol Biol (2000) 74(1–2):37–61. 10.1016/S0079-6107(00)00014-6 11106806

[12] Muller DJ Sapra KT Scheuring S Kedrov A Frederix PL Fotiadis D Single-molecule studies of membrane proteins. Curr Opin Struct Biol (2006) 16(4):489–95. 10.1016/j.sbi.2006.06.001 16797964

[13] Sariisik E Popov C Muller JP Docheva D Clausen-Schaumann H Benoit M. Decoding cytoskeleton-anchored and non-anchored receptors from single-cell adhesion force data. Biophys J (2015) 109(7):1330–3. 10.1016/j.bpj.2015.07.048 26445433 PMC4601042

[14] Chtcheglova LA Hinterdorfer P Simultaneous AFM topography and recognition imaging at the plasma membrane of mammalian cells. Semin Cell Dev Biol (2017). 10.1016/j.semcdb.2017.08.025 28807883

[15] Ebner A Wildling L Kamruzzahan AS Rankl C Wruss J Hahn CD A new, simple method for linking of antibodies to atomic force microscopy tips. Bioconjug Chem (2007) 18(4):1176–84. 10.1021/bc070030s 17516625

[16] Lowndes M Rakshit S Shafraz O Borghi N Harmon R Green K Different roles of cadherins in the assembly and structural integrity of the desmosome complex. J Cell Sci (2014) 127(Pt 10):2339–50. 10.1242/jcs.146316 24610950 PMC4021477

[17] Waschke J Bruggeman P Baumgartner W Zillikens D Drenckhahn D. Pemphigus foliaceus IgG causes dissociation of desmoglein 1-containing junctions without blocking desmoglein 1 transinteraction. J Clin Invest (2005) 115(11):3157–65. 10.1172/JCI23475 16211092 PMC1242188

[18] Baumgartner W Hinterdorfer P Ness W Raab A Vestweber D Schindler H Cadherin interaction probed by atomic force microscopy. Proc Natl Acad Sci U S A (2000) 97(8):4005–10. 10.1073/pnas.070052697 10759550 PMC18132

[19] Priest AV Shafraz O Sivasankar S. Biophysical basis of cadherin mediated cell-cell adhesion. Exp Cell Res (2017) 358(1):10–3. 10.1016/j.yexcr.2017.03.015 28300566

[20] Vielmuth F Wanuske MT Radeva MY Hiermaier M Kugelmann D Walter E Keratins regulate the adhesive properties of desmosomal cadherins through signaling. J Invest Dermatol (2018) 138(1):121–31. 10.1016/j.jid.2017.08.033 28899688

[21] Schlegel N Meir M Heupel WM Holthofer B Leube RE Waschke J. Desmoglein 2-mediated adhesion is required for intestinal epithelial barrier integrity. Am J Physiol Gastrointest Liver Physiol (2010) 298(5):G774–83. 10.1152/ajpgi.00239.2009 20224006

[22] Dieding M Debus JD Kerkhoff R Gaertner-Rommel A Walhorn V Milting H Arrhythmogenic cardiomyopathy related DSG2 mutations affect desmosomal cadherin binding kinetics. Sci Rep (2017) 7(1):13791. 10.1038/s41598-017-13737-x 29062102 PMC5653825

[23] Zhou L Cai M Tong T Wang H. Progress in the correlative atomic force microscopy and optical microscopy. Sensors (Basel) (2017) 17(4):E938. 10.3390/s17040938 28441775 PMC5426934

[24] Hauser M Wojcik M Kim D Mahmoudi M Li W Xu K. Correlative super-resolution microscopy: new dimensions and new opportunities. Chem Rev (2017) 117(11):7428–56. 10.1021/acs.chemrev.6b00604 28045508

[25] Wagner P. Immobilization strategies for biological scanning probe microscopy. FEBS Lett (1998) 430(1–2):112–5. 10.1016/S0014-5793(98)00614-0 9678605

[26] Vielmuth F Waschke J Spindler V Loss of desmoglein binding is not sufficient for keratinocyte dissociation in pemphigus. J Invest Dermatol (2015) 135(12):3068–77. 10.1038/jid.2015.324 26288352

[27] Fung CK Seiffert-Sinha K Lai KW Yang R Panyard D Zhang J Investigation of human keratinocyte cell adhesion using atomic force microscopy. Nanomedicine (2010) 6(1):191–200. 10.1016/j.nano.2009.05.008 19616642

[28] Fung CK Xi N Yang R Seiffert-Sinha K Lai KW Sinha AA. Quantitative analysis of human keratinocyte cell elasticity using atomic force microscopy (AFM). IEEE Trans Nanobioscience (2011) 10(1):9–15. 10.1109/TNB.2011.2113397 21349797 PMC3852989

[29] Vielmuth F Hartlieb E Kugelmann D Waschke J Spindler V. Atomic force microscopy identifies regions of distinct desmoglein 3 adhesive properties on living keratinocytes. Nanomedicine (2015) 11(3):511–20. 10.1016/j.nano.2014.10.006 25510735

[30] Waschke J Spindler V. Desmosomes and extradesmosomal adhesive signaling contacts in pemphigus. Med Res Rev (2014) 34(6):1127–45. 10.1002/med.21310 24549583

[31] Seiffert-Sinha K Yang R Fung CK Lai KW Patterson KC Payne AS Nanorobotic investigation identifies novel visual, structural and functional correlates of autoimmune pathology in a blistering skin disease model. PLoS One (2014) 9(9):e106895. 10.1371/journal.pone.0106895 25198693 PMC4157813

[32] Muller DJ. AFM: a nanotool in membrane biology. Biochemistry (2008) 47(31):7986–98. 10.1021/bi800753x 18616288

[33] Amagai M Karpati S Klaus-Kovtun V Udey MC Stanley JR. Extracellular domain of pemphigus vulgaris antigen (desmoglein 3) mediates weak homophilic adhesion. J Invest Dermatol (1994) 103(4):609–15. 10.1111/1523-1747.ep12397292 7930691

[34] Heupel WM Zillikens D Drenckhahn D Waschke J. Pemphigus vulgaris IgG directly inhibit desmoglein 3-mediated transinteraction. J Immunol (2008) 181(3):1825–34. 10.4049/jimmunol.181.3.1825 18641320

[35] Spindler V Heupel WM Efthymiadis A Schmidt E Eming R Rankl C Desmocollin 3-mediated binding is crucial for keratinocyte cohesion and is impaired in pemphigus. J Biol Chem (2009) 284(44):30556–64. 10.1074/jbc.M109.024810 19717567 PMC2781610

[36] Waschke J Menendez-Castro C Bruggeman P Koob R Amagai M Gruber HJ Imaging and force spectroscopy on desmoglein 1 using atomic force microscopy reveal multivalent Ca^(2+)^-dependent, low-affinity trans-interaction. J Membr Biol (2007) 216(2–3):83–92. 10.1007/s00232-007-9037-9 17657525

[37] Baumgartner W Golenhofen N Grundhofer N Wiegand J Drenckhahn D. Ca^2+^ dependency of N-cadherin function probed by laser tweezer and atomic force microscopy. J Neurosci (2003) 23(35):11008–14. 14657157 10.1523/JNEUROSCI.23-35-11008.2003 PMC6741029

[38] Bell GI. Models for the specific adhesion of cells to cells. Science (1978) 200(4342):618–27. 10.1126/science.347575 347575

[39] Evans E Ritchie K. Dynamic strength of molecular adhesion bonds. Biophys J (1997) 72(4):1541–55. 10.1016/S0006-3495(97)78802-7 9083660 PMC1184350

[40] Harrison OJ Brasch J Lasso G Katsamba PS Ahlsen G Honig B Structural basis of adhesive binding by desmocollins and desmogleins. Proc Natl Acad Sci U S A (2016) 113(26):7160–5. 10.1073/pnas.1606272113 27298358 PMC4932976

[41] Nie Z Merritt A Rouhi-Parkouhi M Tabernero L Garrod D. Membrane-impermeable cross-linking provides evidence for homophilic, isoform-specific binding of desmosomal cadherins in epithelial cells. J Biol Chem (2011) 286(3):2143–54. 10.1074/jbc.M110.192245 21098030 PMC3023511

[42] Amagai M Klaus-Kovtun V Stanley JR. Autoantibodies against a novel epithelial cadherin in pemphigus vulgaris, a disease of cell adhesion. Cell (1991) 67(5):869–77. 10.1016/0092-8674(91)90360-B 1720352

[43] Walter E Vielmuth F Rotkopf L Sardy M Horvath ON Goebeler M Different signaling patterns contribute to loss of keratinocyte cohesion dependent on autoantibody profile in pemphigus. Sci Rep (2017) 7(1):3579. 10.1038/s41598-017-03697-7 28620161 PMC5472593

[44] Vielmuth F Walter E Fuchs M Radeva M Buechau F Magin TM Keratins regulate p38MAPK-dependent desmoglein binding properties in pemphigus. Front Immunol (2018). 10.3389/fimmu.2018.00528 29616033 PMC5868517

[45] Tsunoda K Ota T Aoki M Yamada T Nagai T Nakagawa T Induction of pemphigus phenotype by a mouse monoclonal antibody against the amino-terminal adhesive interface of desmoglein 3. J Immunol (2003) 170(4):2170–8. 10.4049/jimmunol.170.4.2170 12574390

[46] Heupel WM Muller T Efthymiadis A Schmidt E Drenckhahn D Waschke J. Peptides targeting the desmoglein 3 adhesive interface prevent autoantibody-induced acantholysis in pemphigus. J Biol Chem (2009) 284(13):8589–95. 10.1074/jbc.M808813200 19164289 PMC2659217

[47] Payne AS Ishii K Kacir S Lin C Li H Hanakawa Y Genetic and functional characterization of human pemphigus vulgaris monoclonal autoantibodies isolated by phage display. J Clin Invest (2005) 115(4):888–99. 10.1172/JCI24185 15841178 PMC1070425

[48] Spindler V Rotzer V Dehner C Kempf B Gliem M Radeva M Peptide-mediated desmoglein 3 crosslinking prevents pemphigus vulgaris autoantibody-induced skin blistering. J Clin Invest (2013) 123(2):800–11. 10.1172/JCI60139 23298835 PMC3561799

[49] Tsunoda K Ota T Saito M Hata T Shimizu A Ishiko A Pathogenic relevance of IgG and IgM antibodies against desmoglein 3 in blister formation in pemphigus vulgaris. Am J Pathol (2011) 179(2):795–806. 10.1016/j.ajpath.2011.04.015 21718682 PMC3157249

[50] Jennings JM Tucker DK Kottke MD Saito M Delva E Hanakawa Y Desmosome disassembly in response to pemphigus vulgaris IgG occurs in distinct phases and can be reversed by expression of exogenous Dsg3. J Invest Dermatol (2011) 131(3):706–18. 10.1038/jid.2010.389 21160493 PMC3235416

[51] Windoffer R Beil M Magin TM Leube RE. Cytoskeleton in motion: the dynamics of keratin intermediate filaments in epithelia. J Cell Biol (2011) 194(5):669–78. 10.1083/jcb.201008095 21893596 PMC3171125

[52] Seltmann K Fritsch AW Kas JA Magin TM. Keratins significantly contribute to cell stiffness and impact invasive behavior. Proc Natl Acad Sci U S A (2013) 110(46):18507–12. 10.1073/pnas.1310493110 24167274 PMC3832002

[53] Ramms L Fabris G Windoffer R Schwarz N Springer R Zhou C Keratins as the main component for the mechanical integrity of keratinocytes. Proc Natl Acad Sci U S A (2013) 110(46):18513–8. 10.1073/pnas.1313491110 24167246 PMC3831947

[54] Broussard JA Yang R Huang C Nathamgari SSP Beese AM Godsel LM The desmoplakin-intermediate filament linkage regulates cell mechanics. Mol Biol Cell (2017) 28(23):3156–64. 10.1091/mbc.E16-07-0520 28495795 PMC5687018

[55] Berkowitz P Hu P Liu Z Diaz LA Enghild JJ Chua MP Desmosome signaling. Inhibition of p38MAPK prevents pemphigus vulgaris IgG-induced cytoskeleton reorganization. J Biol Chem (2005) 280(25):23778–84. 10.1074/jbc.M501365200 15840580

[56] Waschke J Spindler V Bruggeman P Zillikens D Schmidt G Drenckhahn D.Inhibition of Rho A activity causes pemphigus skin blistering. J Cell Biol (2006) 175(5):721–7. 10.1083/jcb.200605125 17130286 PMC2064672

[57] Gliem M Heupel WM Spindler V Harms GS Waschke J. Actin reorganization contributes to loss of cell adhesion in pemphigus vulgaris. Am J Physiol Cell Physiol (2010) 299(3):C606–13. 10.1152/ajpcell.00075.2010 20554911

[58] Chtcheglova LA Wildling L Waschke J Drenckhahn D Hinterdorfer P. AFM functional imaging on vascular endothelial cells. J Mol Recognit (2010) 23(6):589–96. 10.1002/jmr.1052 21038359

[59] Chtcheglova LA Waschke J Wildling L Drenckhahn D Hinterdorfer P. Nano-scale dynamic recognition imaging on vascular endothelial cells. Biophys J (2007) 93(2):L11–3. 10.1529/biophysj.107.109751 17496017 PMC1896235

[60] Curry N Ghezali G Kaminski Schierle GS Rouach N Kaminski CF. Correlative STED and atomic force microscopy on live astrocytes reveals plasticity of cytoskeletal structure and membrane physical properties during polarized migration. Front Cell Neurosci (2017) 11:104. 10.3389/fncel.2017.00104 28469559 PMC5396045

[61] Santacroce M Orsini F Perego C Lenardi C Castagna M Mari SA Atomic force microscopy imaging of actin cortical cytoskeleton of *Xenopus laevis* oocyte. J Microsc (2006) 223(Pt 1):57–65. 10.1111/j.1365-2818.2006.01596.x 16872432

[62] Usukura E Suzuki T Furuike S Soga N Saita E Hisabori T Torque generation and utilization in motor enzyme F0F1-ATP synthase: half-torque F1 with short-sized pushrod helix and reduced ATP synthesis by half-torque F0F1. J Biol Chem (2012) 287(3):1884–91. 10.1074/jbc.M111.305938 22128167 PMC3265869

[63] Quinlan RA Schwarz N Windoffer R Richardson C Hawkins T Broussard JA A rim-and-spoke hypothesis to explain the biomechanical roles for cytoplasmic intermediate filament networks. J Cell Sci (2017) 130(20):3437–45. 10.1242/jcs.202168 29032358 PMC6518161

[64] Waschke J. The desmosome and pemphigus. Histochem Cell Biol (2008) 130(1):21–54. 10.1007/s00418-008-0420-0 18386043 PMC2413110

[65] Denning MF Guy SG Ellerbroek SM Norvell SM Kowalczyk AP Green KJ. The expression of desmoglein isoforms in cultured human keratinocytes is regulated by calcium, serum, and protein kinase C. Exp Cell Res (1998) 239(1):50–9. 10.1006/excr.1997.3890 9511724

[66] Rotzer V Hartlieb E Vielmuth F Gliem M Spindler V Waschke J. E-cadherin and Src associate with extradesmosomal Dsg3 and modulate desmosome assembly and adhesion. Cell Mol Life Sci (2015) 72(24):4885–97. 10.1007/s00018-015-1977-0 26115704 PMC11113844

[67] Tsang SM Brown L Lin K Liu L Piper K O’Toole EA Non-junctional human desmoglein 3 acts as an upstream regulator of Src in E-cadherin adhesion, a pathway possibly involved in the pathogenesis of pemphigus vulgaris. J Pathol (2012) 227(1):81–93. 10.1002/path.3982 22294297

[68] Oktarina DA van der Wier G Diercks GF Jonkman MF Pas HH. IgG-induced clustering of desmogleins 1 and 3 in skin of patients with pemphigus fits with the desmoglein nonassembly depletion hypothesis. Br J Dermatol (2011) 165(3):552–62. 10.1111/j.1365-2133.2011.10463.x 21692763

[69] Yoshida K Ishii K Shimizu A Yokouchi M Amagai M Shiraishi K Non-pathogenic pemphigus foliaceus (PF) IgG acts synergistically with a directly pathogenic PF IgG to increase blistering by p38MAPK-dependent desmoglein 1 clustering. J Dermatol Sci (2017) 85(3):197–207. 10.1016/j.jdermsci.2016.12.010 28024684 PMC5510496

[70] Stahley SN Warren MF Feldman RJ Swerlick RA Mattheyses AL Kowalczyk AP. Super-resolution microscopy reveals altered desmosomal protein organization in tissue from patients with pemphigus vulgaris. J Invest Dermatol (2016) 136(1):59–66. 10.1038/JID.2015.353 26763424 PMC4730957

[71] Rotzer V Hartlieb E Winkler J Walter E Schlipp A Sardy M Desmoglein 3-dependent signaling regulates keratinocyte migration and wound healing. J Invest Dermatol (2016) 136(1):301–10. 10.1038/jid.2015.380 26763450

[72] Berkowitz P Hu P Warren S Liu Z Diaz LA Rubenstein DS. p38MAPK inhibition prevents disease in pemphigus vulgaris mice. Proc Natl Acad Sci U S A (2006) 103(34):12855–60. 10.1073/pnas.0602973103 16908851 PMC1568937

[73] Berkowitz P Diaz LA Hall RP Rubenstein DS Induction of p38MAPK and HSP27 phosphorylation in pemphigus patient skin. J Invest Dermatol (2008) 128(3):738–40. 10.1038/sj.jid.5701080 17928890

